# Sensitization patterns to Poaceae pollen indicates a hierarchy in allergens and a lead of tropical grasses

**DOI:** 10.1002/clt2.12287

**Published:** 2023-08-07

**Authors:** Victoria Rodinkova, Serhii Yuriev, Vitalii Mokin, Olena Sharikadze, Yevhenii Kryzhanovskyi, Lilia Kremenska, Olha Kaminska, Andrii Kurchenko

**Affiliations:** ^1^ Department of Pharmacy National Pirogov Memorial Medical University Vinnytsya Ukraine; ^2^ Department of Clinical Immunology and Allergology Bohomolets National Medical University Kyiv Ukraine; ^3^ Medical Centre DIVERO Kiev Ukraine; ^4^ Department of System Analysis and Information Technologies Vinnytsia National Technical University Vinnytsia Ukraine; ^5^ Paediatric Department Shupyk National Healthcare University Kyiv Ukraine

**Keywords:** climate change, component‐resolved allergy diagnostics, grass pollen groups I, II/III, V, VI, grass pollen sensitization, statistical inference

## Abstract

**Background:**

The allergenicity of pollen of Poaceae family members is a well‐known and confirmed fact. Using the data of component‐resolved molecular diagnostics of allergy, we set a goal to establish the population and individual characteristics of sensitization to grass pollen and assess the patterns of its development.

**Methods:**

Multiplex allergy Alex^2^ test results of 20,033 patients were used. In addition to descriptive statistics to uncover traits of the sensitized population, statistical inference was utilized to establish the conditional probability of sensitisation, the nature of links between allergens, and the most frequent combinations of allergens in individual patient profiles.

**Results:**

Sensitivity to grass pollen comprised 30.79% of the studied sample. Children accounted for 62.21%, adults—37.79%. Sensitisation to Phl p 1, Lol p 1, and Cyn d 1 was the most frequent in all age groups. Among them, Phl p 1 and Lol p 1 were the major ones. Phl p 2, Phl p 5.0101, and Phl p 6 were also responsible for primary sensitization; Phl p 5.0101 promoted the highest sIgE levels. A combination “Lol p 1—Phl p 1”, where Lol p 1 might play a leading role, was most frequent in individual profiles. Monosensitization to Phl p 2 was the second most frequent and Bayesian Network suggested its independent development. Monosensitization to Cyn d 1, especially among children, may indicate the impact of climate change, promoting the spread of the subtropical grasses to the temperate region.

**Conclusions:**

Descriptive statistics and known clinical data coincide well with statistical inference results and can provide for new clinical insights.

## 1 INTRODUCTION

The allergenicity of pollen from plants of the Poaceae family is a well‐known and confirmed fact.^1^, ^2^ Depending on the climate and regional characteristics, sensitivity to this type of pollen can reach up to 30%.^3^ Exposure to Poaceae pollen causes symptoms of allergic rhinoconjunctivitis and/or asthma in sensitive individuals,^4^ such as thunderstorm asthma in Australia.^5^ In addition, even short‐term exposure to grass pollen can cause changes in reactivity to skin prick tests; it can influence the course of food allergies and exacerbations of eczema in children.^6^

The so‐called group I allergens, which include beta‐expansins,^7^ in particular Phl p 1, were identified earlier as a marker of sensitization to grass pollen. However, recent data indicate that the IgE to group II/III and group V grass allergens, such as Phl p 2 or Phl p 5.0101, respectively, can also serve as a marker of genuine sensitivity of patients to Poaceae pollen.

Clinical data indicate that the development of grass pollen sensitization occurs from the monomolecular stage to the polymolecular one through oligomolecular sensitization.^8^ Other data suggest that polysensitization to Poaceae pollen develops from childhood.^9^ Because of polysensitization, as well as the high cross‐reactivity of Poaceae pollen, especially in temperate climate,^10^ determining the causative allergens for a specific patient is difficult since the pollen seasons of different types of grasses overlap in time.^11^ In addition, the pollination of plants of this family can last up to several months in a temperate climate. In subtropical countries, they can even be in flower year‐round.^12^, ^13^ ^,^

The diagnosis and prevention of sensitization to Poaceae pollen are also complicated by the geographical distribution of individual grass species and climate change favoring the spread of subtropical grasses, such as *Cynodon*, in temperate climates.^14^ In addition, it is impossible to clearly determine the pollination season of different grasses during aerobiological studies^15^ due to the similar morphology of their pollen.^16^

This, in turn, results in a special pattern of sensitization to Poaceae pollen in each population. Moreover, the list of species in the Poaceae family that can cause allergies is constantly expanding and currently it includes the pollen of 21 types of grasses.^17^ Hence, all the above factors indicate the importance of grass pollen as an airborne allergen.

In case of polymorphism of allergic triggers in the Poaceae family and polysensitization to them, the use of component‐resolved molecular allergodiagnostics seems to be the optimum.

Thus, the aim of the presented work was to perform a comprehensive analysis of the sensitization profile to grass pollen in the Ukrainian population with the determination of both population and individual sensitivity to grass allergens, possible interrelations between culprit allergens in their ability to promote this sensitivity and patterns of grass sensitization changes in people of different age groups.

## 2 METHODS

### 2.1 Participants

To solve the problem set, we used the data of 20,033 people aged from 1 to 89 years who lived in 17 different regions of Ukraine and who were tested by the multiplex allergy test ALEX^2^ during 2020–2022.

Patients with a history of allergic rhinitis and/or atopic dermatitis and/or bronchial asthma were included in the study. Non‐inclusion criteria comprised absence of allergy history and absence of sensitization to grass pollen.

The decision to prescribe component‐resolved molecular allergy diagnostics was made by the doctor to whom the patient presented with the symptoms of the above‐mentioned diseases and was based on the protocols of the Ministry of Health of Ukraine for the diagnosis and treatment of allergic diseases. These include Unified clinical protocol of primary, secondary (specialized) medical aid for Asthma^18^ Unified clinical protocol of primary, secondary (specialized), and tertiary (highly specialized) medical aid for Atopic Dermatitis^19^; Evidence‐based clinical guidelines on Asthma^20^ ARIA Guidelines on Allergic Rhinitis and its Impact on Asthma (2016 Revision) approved in Ukraine at an extended meeting of state experts in accordance with Orders of the Ministry of Health of Ukraine.^21^

Test results from ALEX^2^ obtained at the Medical Centre, DIVERO, Kiev, Ukraine were considered. The data were kindly provided by “Alex Diagnostics Ukraine LLC” which is the exclusive dealer of the Alex^2^ test in our country. In total, 45,700 Alex^2^ tests were performed in Ukraine during the study period and we analyzed data from 43.84% of these tests.

### 2.2 Ethics

All patients signed informed consent before testing. Among others, it included a paragraph about the possible usage of impersonalized patient data for scientific purposes. The Commissions for Bioethical Expertise and Research Ethics at the Bogomolets National Medical University, Kyiv, Ukraine, approved the study protocol. In this study, we did not aim to collect and analyze detailed symptomatology of patients in the context of its correspondence with the molecular test results. Instead, the main attention was paid to the analysis of epidemiological patterns of regional, individual, and age sensitization to grass pollen in the Ukrainian population and the factors that can influence it.

### 2.3 Variables and data sources

The components of multiplex allergy test ALEX^2^ used to determine the sensitization of each patient were the following: molecule from the group of beta‐expansins Cyn d 1 of Bermuda grass (*Cynodon dactylon*) and Cyn d extract; beta‐expansin molecule Lol p 1 of perennial ryegrass (*Lolium perenne*); allergen extracts Pas n of bahiagrass (*Paspalum notatum*), Phr c of common reed (*Phragmites communis*), Sec c_pollen extract of rye (*Secale cereale*); as well as a number of Timothy grass (*Phleum pratense*) molecules. These were, in particular, beta‐expansin Phl p 1; allergen Phl p 2 of grass group II/III; isoallergen of group V Phl p 5.0101; P‐particle‐associated protein of grass group VI Phl p 6; polcalcin Phl p 7 and profilin Phl p 12. The threshold level was set at 0.31 kU/L, according to the reference values of the ALEX^2^ test.

The sex, age, and medical conditions of the patients were also recorded (Table 1). To determine the IgE‐reactivity pattern to grass pollen in different age groups, we assessed sensitivity patterns in the following age groups: 0–1 year, 2–3, 4–6, 7–12, 13–18, 19–25, 26–36, 37–44 and 45–60 years. Stratification of children's age (up to 18 years) was based on the generally accepted classification, which includes the infant period up to 1 year, toddler 1–3 years, preschool child up to 6, primary school age (6–12 years), high school age or puberty (12–18 years). The selected age values of the adults corresponded to the age of onset and end of youth (25 and 44 years, respectively) and the end of middle age (60 years).^28^ In addition, the age of 36 years was taken for the analysis as an intermediate.

**TABLE 1:** Specifics of reference group sensitive to grass pollen components in the studied sample.

Indicators	Values
Sex	
Male	3403 (55.2%)
Female	2766 (44.8%)
Area of residency in Ukraine	
Central	1427 (23.1%)
Eastern	802 (13.0%)
Western	1077 (17.5%)
Southern	1094 (17.7%)
Northern	1769 (28.7%)
Age	
1–8 years	1872 (30.3%)
9–17 years	1966 (32.0%)
18–24 years	569 (9.2%)
25–32 years	610 (9.9%)
33–40 years	616 (9.9%)
41–48 years	280 (4.5%)
49–56 years	155 (2.5%)
57–64 years	85 (1.4%)
>65 years	16 (0.3%)
Condition	
Asthma	274 (4.4%)
Recurrent wheezing	1727 (28.0%)
Rhinitis	2838 (46.0%)
Dyspnea	187 (3.0%)
Atopic dermatitis	624 (10.1%)
Family history of asthma	879 (14.2%)

### 2.4 Data analysis

General characteristics of the sample population, namely, the distribution of patients by age, by the level of specific IgE (sIgE) in the tested sera to the above‐mentioned components of grass pollen, and by levels of sensitivity to these components in individual age groups were calculated using descriptive statistics of MS Excel 2013.

In other cases, we applied approaches of inferential statistical analysis, which allows generalizing about the population when only a sample is available.^22^

To determine combinations of allergenic components in individual profiles of patients, a complex of programs in the Python language was developed. Bayesian network (BN) analysis, a key tool in statistical inference,^22^ was used to determine the probability of developing combined sensitization in individual patients and molecular components of which might play a leading role. It was chosen since BN analysis makes it possible to establish the probability of certain processes being related to the health of the population.^9^

A BN is a Directed Acyclic Graph (DAG). A graph is acyclic if it has no paths that start and end at the same node. A graph is directed if arrows (edges) connect nodes. Their direction reflects which node affects another one. The BN makes it possible to determine the so‐called parent root nodes, which influence other nodes dependent on them.^23^ Thus, each DAG allows drawing certain conclusions about the probability of the occurrence of some events, depending on the probability of others. Therefore, the connections found show the probability of developing sensitization to some allergens depending on the sensitivity to others based on the interconnected influence of factors.

In this study, the root node for DAG was set to Phl p 1 as for the genuine allergen of primary importance in grass sensitization. The values of the conditional probability distributions (CPD) of interconnections between allergens in the individual patient profiles were calculated for each studied component.

To determine regional characteristics of sensitivity to grass pollen in each particular region, the frequency of patients with sensitivity to each of the molecules was calculated separately. Then, the priority molecules for the region were determined. If the frequency of the sensitisation to the first (absolute) maximum exceeded the frequency of the next one by more than 5%, then only the molecule with the top sensitivity was considered as the main agent in the region. If the frequency of sensitization to some components differed by less than 5%, then it was concluded that the residents of the region were sensitized to two or more molecules at the same time. ArcGIS Maps SDK for JavaScript was used to build a map.

## 3 RESULTS

### 3.1 Characteristics of the patients

Sensitization to at least one Poaceae allergen was found in 6169 people, which was 30.79% of the studied population. The percentage of sensitized children under the age of 18 (62.21%) was 1.65 times higher than that of adults (37.79%).

The distribution patterns of the variables in the dataset were away from normal (See Supplementary material S2), and the median age of the Poaceae‐sensitized group was 13 years. The median age of children was 9 years, of adults—32 years. The group of children aged 9–17 was the largest in the entire sample. The children's group was dominated by 7‐8 year‐olds. Among the adults, the age group of 25–40 years prevailed (Table 1).

### 3.2 Specifics of sensitization to Poaceae allergens in the study sample

The sensitization profile of the studied population showed that IgE‐reactivity to Phl p 1 was the most frequent one, followed by sensitization to Lol p 1 and Cyn d 1. Sensitivity to Phl p 1 was observed either alone or in combination with other allergenic molecules in 63.79% of patients. The percentage of people sensitized to other beta‐expansins Lol p 1 and Cyn d 1 was also significant (56.09% and 46.07%, respectively). The percentage of those sensitized to beta‐expansins was the highest in both children and adults, separately. The percentage of the patients sensitive to polcalcin Phl p 7 was the lowest. It is notable that the percentage of patients sensitized to allergen group II Phl p 2 was higher in children than in adults (30.59% vs. 25.65%); similar results were found comparing sensitization of children and adults to Phl p 5.0101 (20.77% vs. 30.03%) and to Phl p 6 (13.81% vs. 22.01%).

The largest number of monosensitized patients was observed for profilin Phl p 12, which is not a marker of genuine sensitization to grass pollen. Therefore, Phl p 2 was in the first place in terms of true sensitivity among the monosensitized people of the studied sample. Monosensitivity to Phl p 1 was the second and Cyn d 1 was third. Moreover, the number of patients monosensitized to Phl p 2 and Cyn d 1 significantly prevailed among children. Monosensitivity to Phl p 1 held the third place for children. In adults, the levels of monosensitivity to Phl p 1 and Phl p 2 remained the highest. Among the extracts, sensitivity to Cyn d was the most frequent, followed by Sec c_pollen extract in all age groups.

Phl p 5.0101 was the allergen that induced the highest median sIgE levels. They were sharply higher than sIgE‐s in any other group. Phl p 1 was second in all age categories, and Lol p 1 and Phl p 6 were in the third and fourth place in the general group and in the group of adults, respectively. Phl p 6 was third in the group of children followed by Phl p 12. Lol p 1 was just the fifth one in this case.

In most cases, sIgE levels decreased in adults. However, for Phl p 2 and Phl p 7 an increase in the average value of sIgE was observed.

Phl p 1 and Lol p 1 could be referred to as major allergens in our sample as sensitivity to these two molecules exceeded 50% among both adults and children. The sensitivity to both the extract and beta‐expansin of *Cynodon dactylon* approached 50%, but did not exceed this limit (Table 2).

**TABLE 2:** Number, percentage and median sIgE values of patients sensitized to molecular allergens of grass pollen.

Name of the allergenic component/biochemical name	Median/IQR of slgE in sensitive individuals, kU/L	Number (%) of patients sensitive to the allergen	Number (%) of patients monosensitised to the allergen	Median/IQR of slgE in the group of children, kU/L	Number (%) of children sensitive to the allergen	Number (%) of children monosensitised to the allergen	Median/IQR of slgE in the group of adults, kU/L	Number (%) of adults sensitive to the allergen	Number (%) of adults monosensitised to the allergen
Cyn d/Extract	1.80/4.58	2571 (41.68)	9 (0.15)	1.91/5.20	1557 (40.57)	4 (0.10)	1.74/3.78	1014 (43.50)	5 (0.21)
Cyn d 1/Beta‐expansin	2.52/6.36	2842 (46.07)	194 (3.14)	2.59/7.23	1734 (45.18)	171 (4.46)	2.48/5.26	1108 (47.53)	23 (0.99)
Lol p 1/Beta‐expansin	5.71/16.60	3460 (56.09)	12 (0.19)	6.08/20.95	1953 (50.89)	4 (0.10)	5.25/11.34	1507 (64.65)	8 (0.34)
Pas n/Extract	1.12/1.97	1627 (26.37)	14 (0.23)	1.23/2.29	995 (25.92)	5 (0.13)	0.96/1.74	632 (27.11)	9 (0.39)
Phl p 1/Beta‐expansin	7.01/22.88	3935 (63.79)	296 (4.80)	7.33/27.63	2239 (58.34)	161 (4.19)	6.73/18.37	1696 (72.76)	135 (5.79)
Phl p 2/Grass group II/III	1.61/6.11	1772 (28.72)	436 (7.07)	1.18/2.86	1174 (30.59)	365 (9.51)	3.48/8.58	598 (25.65)	71 (3.05)
Phl p 5.0101/Grass group V	19.36/32.61	1497 (24.27)	85 (1.38)	21.62/35.93	797 (20.77)	62 (1.62)	17.83/28.56	700 (30.03)	23 (0.99)
Phl p 6/ grass group VI	5.29/12.62	1043 (16.91)	12 (0.19)	6.67/20.94	530 (13.81)	10 (0.26)	4.40/8.09	513 (22.01)	2 (0.09)
Phl p 7/Polcalcin	1.29/6.10	153 (2.48)	41 (0.66)	1.08/4.46	110 (2.87)	26 (0.68)	2.89/26.6	43 (1.84)	15 (0.64)
Phl p 12/Profilin	5.12/8.94	1730 (28.04)	530 (8.59)	6.58/14.07	1094 (28.50)	284 (7.40)	3.21/4.92	636 (27.28)	246 (10.55)
Phr c/Extract	0.66/0.90	859 (13.92)	22 (0.36)	0.71/1.03	572 (14.90)	11 (0.29)	0.57/0.70	287 (12.31)	11 (0.47)
Sec c_pollen/Extract	2.07/5.02	1951 (31.63)	59 (0.96)	2.20/5.89	1092 (28.45)	36 (0.94)	1.89/4.11	859 (36.85)	23 (0.99)

*Note*: The average level of tIgE for the entire Poaceae‐sensitized group was 352.42 ± 489.15 kU/L. The average level of tIgE in children was 423.79,23 ± 534.89 kU/L, the average level of tIgE in adults—226.33 ± 369.53 kU/L.

Abbreviation: IQR, Interquartile range.

**FIGURE 1:**
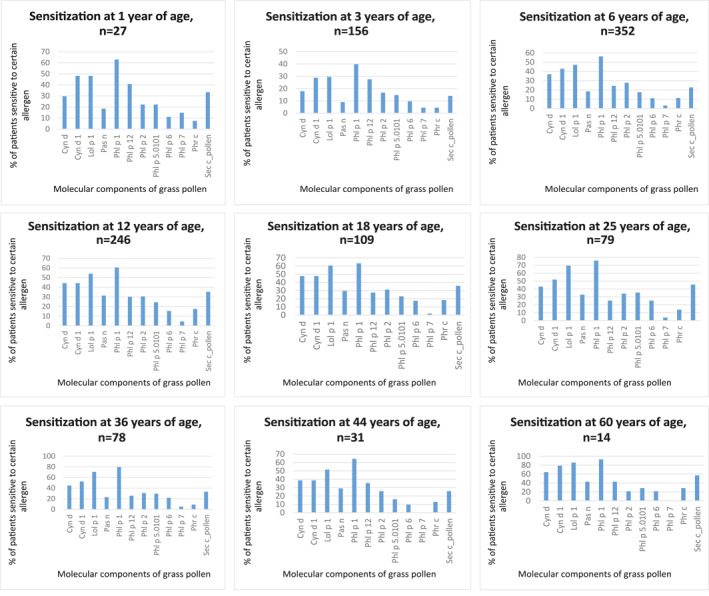
Specifics of sensitization to grass pollen among patients of different age groups in Ukraine.

The highest percentage of patients in each group, starting from the age of 1 year, was sensitized to beta‐expansins Phl p 1, Lol p 1 and Cyn d 1 with a predominance of sensitization to Phl p 1. Sensitivity to Cyn d and Sec c_pollen prevailed among the extracts. The general pattern of sensitization was approximately the same for each age group (Figure 1).

**FIGURE 2:**
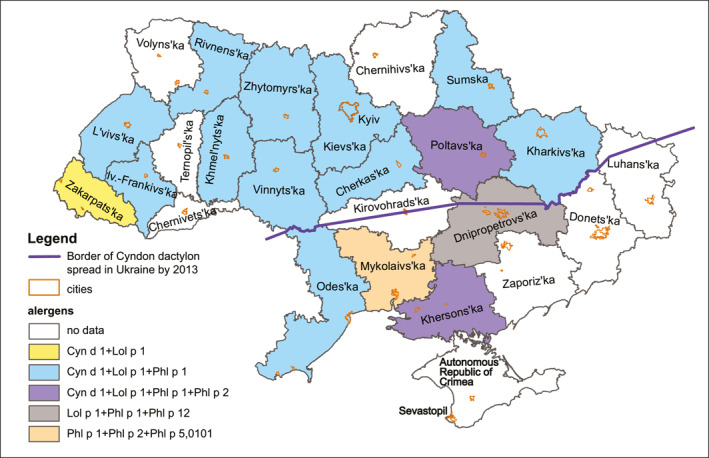
Specifics of regional sensitivity to molecular components of grass pollen in Ukraine.

A similar pattern of sensitization with a dominance of sensitivity to the three beta‐expansins Cyn d 1, Lol p 1 and Phl p 1 was observed in 11 of 16 regions of Ukraine for which sensitization data were available. Phl p 2 was added to these three allergens in the Poltava and Kherson regions. Lol p 1, Phl p 1 and Phl p 12 were the most significant in the Dnipropetrovsk region. Sensitivity to Cyn d 1 and Lol p 1 prevailed in Zakarpattya. In the Mykolaiv region, sensitivity to Phl p 1, Phl p 2, Phl p 5.0101 was the most frequent (Figure 2).

While determining the combinations of molecular allergens that were most often found in individual profiles, it was established that the largest number of patients were sensitized to Phl p 1 and Lol p 1 only. Patients sensitized to profilin Phl p 12 held the second place, and monosensitization to Phl p 2 was the third. Among 26 children aged 3 years who were sensitive to Phl p 2 in our sample, 19 or 73.1% were monosensitized to Phl p 2. Another four had simultaneous sensitivity to group 1 allergens.

**FIGURE 3:**
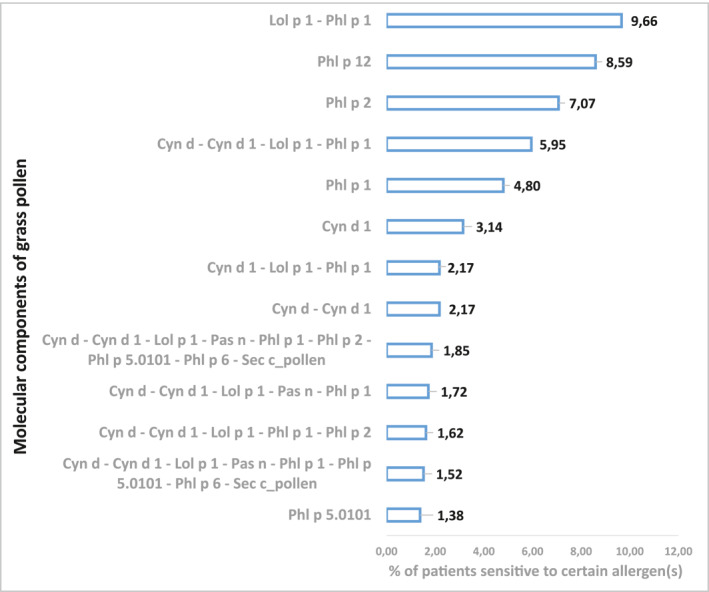
Percentage of people sensitized to different combinations of molecular allergens of grasses.

Simultaneous sensitization of patients to Cyn d 1, Lol p 1, Phl p 1 and Cyn d extract was next in terms of frequency. Simultaneous sensitization only to beta‐expansins Cyn d 1, Lol p 1, Phl p 1 was the seventh most frequent. In addition to monosensitization to Phl p 2, the top 10 most common combinations included monosensitization to Phl p 1 (4.80%) and Cyn d 1. Monosensitization to Phl p 5.0101 was at position 13 (Figure 3, Supplementary material S3).

Interestingly, among 94 or 60.3% of 3‐year‐old children who did not have an elevated level of sIgE to Phl p 1 in our sample, no one was also sensitized to Lol p 1. However, sensitivity to Cyn d 1 was detected in 18 of them. Among 6‐year‐old children, 153 of them or 43.5% did not have antibodies to Phl p 1. Only 4 of them were sensitized to Lol p 1, but 35 were sensitized to Cyn d 1. Moreover, 2 children were sensitive to Cyn d 1 and Lol p 1 simultaneously. In addition, the level of IgE to the first exceeded the level of IgE to second one (2.04 kU/L vs. 0.36 kU/L and 1.53 kU/L vs. 0.55 kU/L, respectively), which confirms the leading role of Cyn d 1 in the induction of sensitivity to grasses.

### 3.3 The analysis of probabilistic connections between sensitization to different grass molecular components using Bayesian network analysis

The analysis of the BN data made it possible to determine that Lol p 1 can play a leading role in the combination Lol p 1—Phl p 1, which occurred most often. It determined sensitization to Phl p 1 together with Cyn d 1, Phl p 2 molecules and Sec c_pollen extract. Conditional probability distributions showed that simultaneous sensitization to Phl p 1, Lol p 1, Cyn d 1, Phl p 2 and Sec c_pollen was possible in 94.7% of cases. Sensitivity to Lol p 1, for its part, was most influenced by Cyn d 1, Pas n, Phl p 2 and Phl p 5.0101. CPD of simultaneous sensitization to them was estimated at 92.0%.

Along with descriptive statistics (Figure 3), Bayesian modeling showed that the development of sensitization to Phl p 2 is independent and unrelated to sensitization to other allergens. Conditional probability distributions of sensitization to this allergen was 32.5%. In turn, Phl p 2 was the only allergen that influenced the development of sensitivity to Phl p 6. Interestingly, the CPD in the absence of simultaneous sensitization to both allergens was the highest—83.5%. Both allergens—Phl p 2 and Phl p 6—influenced the development of sensitivity to rye extract. CPD of simultaneous sensitization to all three agents was 79.9%.

**FIGURE 4:**
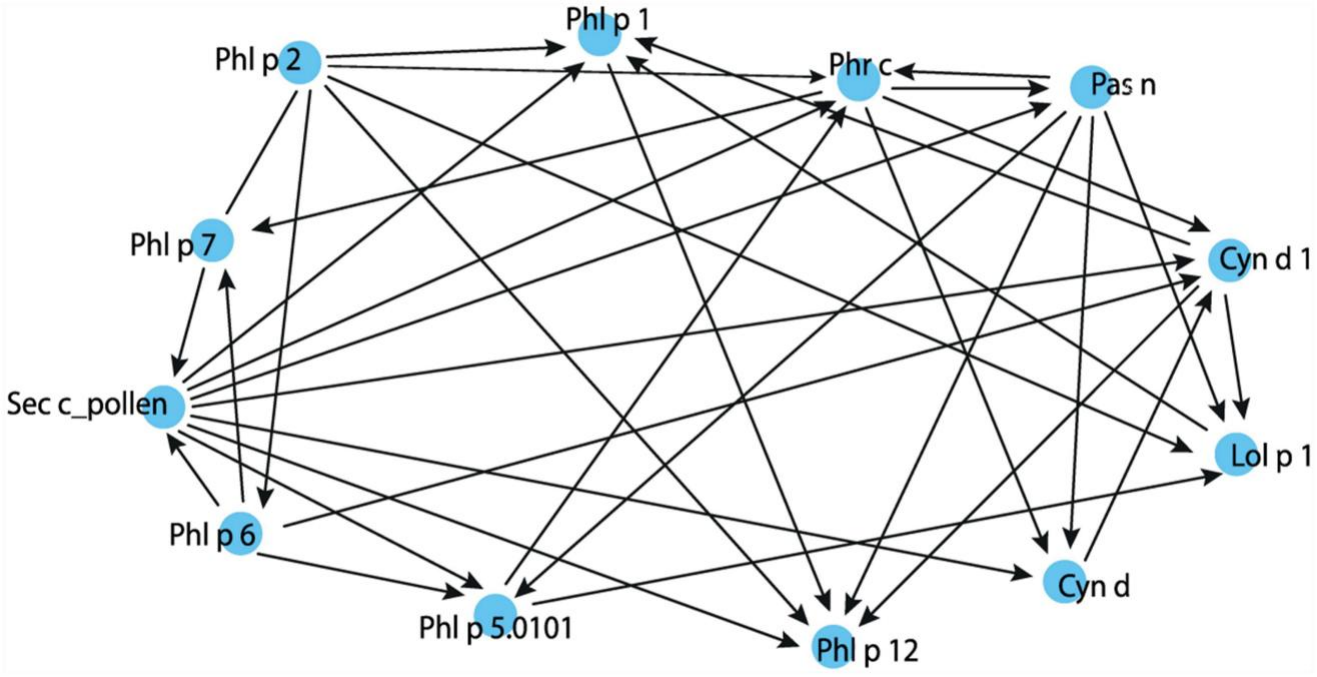
The resulting Bayesian Directed Acyclic Graph (DAG) of probabilistic connections between allergenic components of grasses in the individual patient profiles of the studied sample.

In addition, Phl p 6 affected the sensitivity to Phl p 5.0101 and Phl p 7. Together with extracts of rye and *Paspalum*, they influenced Phl p 5.0101, together with Phr c—Phl p 7. Moreover, the connection between Phl p 5.0101 and Phl p 6 was direct: simultaneous sensitization to these two allergens as well as to Pas n and Sec c_pollen extracts was possible in 89.2% (Figure 4). Conditional probability distributions between Phl p 6 and Phl p 7 was inverse: simultaneous sensitization to them and to Phr c was probable only in 33.3% (See Appendices for per cent of CPD).

## 4 DISCUSSION

Our data are consistent with literature sources,^8^ and they indicate that sensitivity to grass pollen components can progress to the polymolecular stage. However, the initial molecule may be different and therefore it is important to use a strategy of early diagnosis and possibly consider a correct age for allergen immunotherapy.^9^

The novelty of the present study lies in the fact that BN analysis could be an effective tool able to establish the hierarchy of sensitization to certain allergens during the transfer from mono‐to oligo‐ and polysensitization. Thus, by controlling the sensitivity to the key “parental” allergen, it is potentially possible to reduce the subsequent sensitization or even prevent it.

To our knowledge, this is the first paper in the world which uses a BN in order to establish the hierarchy in the sensitization to grass allergens. Previous papers used BN as a decision support system determining the skin^24^, ^25^ (and house dust mite sensitization).^26^ However, application of Bayesian modelling for the healthcare systems seems to be useful and needs further clinical adoption^27^

This was shown in this study as many of the patterns found by the BN are consistent with the known clinical data. For example, modelling suggested that while affecting the development of sensitization to other allergens, Phl p 2 itself was not affected by them. This can explain the significant number of patients monosensitized to Phl p 2 in the population. This is entirely consistent with the data that group II/III grass allergens act as independent inducers for allergic sensitization.^10^ According to Bayesian modelling, Phl p 2 also played a leading role in the induction of IgE‐antibody formation in both Lol p 1 and Phl p 1 and this idea needs further clinical examination. In the pair Lol p 1—Phl p 1, which was the most common combination in individual patient profiles, the leading role of Lol p 1 can be explained by the greater ability of *Lolium perenne* to adapt to arid conditions^28^ and its wider distribution on the territory of Ukraine compared to Timothy grass.^29^ Interestingly, apart from Phl p 2, sensitization to Lol p 1 was determined by Cyn d 1 and Phl p 5.0101, which are also considered genuine markers of sensitization to grass pollen.^10^

The fact that the most frequent sensitization in the Ukrainian population was observed to group I grass allergens Lol p 1 and Phl p 1is well consistent with literature discussing the high frequency of cross‐sensitization to these allergens.^30^, ^31^, ^32^, ^33^

A significant percentage of those monosensitized to Cyn d 1 may indicate a new trend for monosensitization in Ukraine, especially in children, to a subtropical grass such as *Cynodon dactylon* (Bermuda grass). These species is native to tropical, subtropical and partially temperate regions of the Old World.,^7^ and according to the literature, it also occurs in Ukraine, which is in a temperate zone; it is naturally distributed in the south of the steppe zone and in the Crimea, but can spread passively by rail to the northern regions.^34^ It was reported in 2013 that the northern border of the modern distribution of *Cynodon dactylon* in Ukraine ran along approximately the following line: the border of Vinnytsia and Odesa regions—Dnipropetrovsk—somewhat north of Donetsk and further to the east^35^ (Figure 2). At the same time, according to our data, monosensitization to *Cynodon dactylon* both in adults and in children, the youngest of whom was 2 years old, was observed in Khmelnytskyi, Vinnytsia, Ivano‐Frankivsk, Kyiv, Lviv, Rivne, Sumy, Zakarpattia, Kharkiv regions, which are located further north of the delineated border. It is known from recent literature sources that in the forest‐steppe zone of Ukraine, *C*. *dactylon* forms colonies that gradually widen their area due to intensive vegetative reproduction, forming dense, almost monodominant thickets. Such a colony was recorded for the first time on August 7, 2018 on the Left Bank of the Kyiv region.^34^

The rapid development of sensitivity to *C*. *dactylon* may be related to the high productivity of this herb, which forms considerable quantity of productive stems.^36^

In addition, the fact established in this paper that Cyn d 1 was a parental allergen in relation to Lol p 1 and Phl p 1 may support the idea of low cross‐reactivity between group 1 pollen allergens of subtropical and temperate grasses^37^ and can indicate that sensitization to Cyn d 1 may precede the sensitization to Lol p 1 and Phl p 1 promoting it in the future.

R. E. Rossi et al, 2008^32^ draw attention to co‐sensitization between Bermuda grass allergens and Timothy grass molecules. According to their data, more than 72% of Bermuda grass allergic patients were co‐sensitized to rPhl p 1, rPhl p 2, nPhl p 4, rPhl p 5.0101, and rPhl p 6.

Data from our modelling suggest that the development of sensitivity to Phl p 6 was negatively influenced only by Phl p 2. This may indicate that they could be independent triggers and markers of true sensitization to Timothy grass pollen.^10^

The highest IgE level promoted by Phl p 5.0101 can also serve as a marker of genuine grass sensitization, which is consistent with previous data.^10^ Sensitivity to it observed in each age group is also consistent with literature sources.^33^ A decrease in sensitivity and IgE levels with age also correlates with earlier literature.^32^

In favor of the idea that Phl p 5.0101 could be a genuine allergen, there is the fact that sensitivity to it, according to Bayesian modelling, was associated only with sensitization to the Phl p 6 molecule. Also, Phl p 5.0101 belongs to the same biochemical family. In addition, Phl p 6 is known for its high cross‐reactivity with Phl p 5.0101 and does not add a lot of diagnostic information once IgE to Phl p 5.0101 has been documented.^10^ However, in our case, Phl p 6 was the parental node in relation to Phl p 5.0101. This may indicate its independent role in the formation of sensitization to grass pollen and requires further research.

Despite our data and data from the literature, which lists Phl p 1, Phl p 2, Phl p 5.0101, and Phl p 6^38^ as major grass allergens, in our study percentages of sensitivity to these allergens were much lower than those described in other sources, where sIgE to Phl p 1 was found in 99% of patients, to rPhl p 5.0101—in 67% of patients, to rPhl p 7—in 5%.^33^

The level of sensitization to Phl p 6 (16.91%) in our sample was also much lower compared to other data where the sensitization was up to 75%.^39^ The frequency of sensitization to Phl p 2 was about half of that described earlier in contrast to around 60%–80% recorded in European grass pollen allergic patients.^10^ However, in China none of the patients had sIgE to Phl p 2. Instead, the level of sensitivity to Phl p 1 was 22%, to Phl p 5.0101%–14%, to Phl p 6%–8% and to Phl p 7%–3%.^40^ From these data, only the result for Phl p 7 coincides with those obtained in the current study. However, the sensitivity to this component, as it was already noted, is non‐essential. Thus, patterns of IgE cross‐reactivity to grass pollen appear to depend on the geographical region of the patient population being investigated.^41^

Limitations of the presented study include the lack of analyses of the clinical data from every patient and comparison of the symptoms with the sensitization patterns. On the other hand, it was not the aim of our study which was rather population based. Furthermore, the inclusion of the clinical data could significantly complicate the analyses and comprehension of the obtained results. We consider them sufficiently evident as the obvious strength of our study is the inclusion of data from more than 20,000 patients tested with component‐resolved diagnostics, which includes significant numbers of grass components. Another important advantage of the presented work is the usage of the statistical inference approach when the data are computed regardless of personal intervention.

## 5 CONCLUSIONS

Sensitization to Phl p 1, Lol p 1, Cyn d 1 was the most frequent in the sample. Only Phl p 1 and Lol p 1 belong to the major allergens. Analysis indicated that Phl p 2, Phl p 5.0101 and Phl p 6 were genuine markers of grass pollen sensitization along with mentioned molecules.

By analyzing the molecular sensitization profile in patients with grass pollen allergy from Ukraine, we found that sensitization to Phl p 1, Lol p 1 and Cyn d 1 occurred most frequently. Phl p 2, Phl p 5.01.01 and Phl p 6 were also genuine markers of grass pollen sensitization in our cohort.

A combination Lol p1—Phl p 1, where Lol p 1 might play a leading role, was the most frequent in individual profiles. Sensitivity to Phl p 2 was the second most frequent and BN analysis indicates its independent development.

Monosensitization to Cyn d 1 may suggest the impact of climate change, which promotes the spread of subtropical grasses to the temperate region and leads to changes in the character of sensitization, as Bayesian modelling places Cyn d 1 to the parental position in relation to allergens of group I of temperate grasses.

Descriptive statistics and published clinical data were a good match with the data of statistical inference. The latter make it possible to establish how sensitization to some allergens can be associated with sensitization to others so that inferential statistical analysis can become the basis of the search for new clinical patterns.

## AUTHOR CONTRIBUTIONS

**Victoria Rodinkova**: Formal analysis; conceptualization; writing original draft; review & editing. **Serhii Yuriev**: Conceptualization; data curation; funding and other resources acquisition; investigation; project administration; writing original draft; review & editing. **Vitalii Mokin**: Methodology; software; data validation and analyses; review & editing. **Olena Sharikadze**: Conceptualization; data curation; writing original draft; review & editing. **Yevhenii Kryzhanovskyi**: Formal analysis; methodology and visualization. **Lilia Kremenska**: Conceptualization; discussion the results and writing original draft. **Olha Kaminska**: Descriptive statistics; writing original draft. **Andrii Kurchenko**: Conceptualization; data curation; formal analysis; methodology; project administration and supervision.

## CONFLICT OF INTEREST STATEMENT

All authors declare that the research was conducted in the absence of any commercial or financial relationships that could be construed as potential conflicts of interest.

## Supporting information

Supporting Information S1

Supporting Information S2

Supporting Information S3

## Data Availability

Most data that support the findings of this study (excluding personal data of patients) are available in the supplementary material of this article and via the link provided in the Manuscript; primary patients' data can be shared particularly following email requests to the corresponding author due to potential sensitivity of the data of individual patients.

## Appendix

Principles of BN construction and their results for the given dataset can be seen at https://www.kaggle.com/code/vbmokin/pollen‐bn‐for‐paper.

Descriptive Statistic analyses applied to the studied dataset is provided in the separate file.

Five hundred thirty‐eight Primary patients’ data can be shared particularly just following email request to the 539 corresponding author due to potential sensitivity of the data of individual patients.
