# Supplementary material for: Sensitization patterns to Poaceae pollen indicates a hierarchy in allergens and a lead of tropical grasses
Source: Clin Transl Allergy. 2023 Aug 7;13(8):e12287. doi: 10.1002/clt2.12287 (PMC10405149; doi:10.1002/clt2.12287)
Supplement: Supplementary file 1 — Supporting Information S1 [file CLT2-13-e12287-s002.docx]

Caption for the Suppl_Grass_Pollen_Alex_20333_Descriptive_Statistics is:

Descriptive statistics for Poaceae-sensitized patients’ dataset obtained with the multiplex allergy test ALEX2 in Ukraine during 2020-2022.

Caption for the Supplement_Statistical_Inference_in_Grass_Sensitization is as follows:

Python-generated combinations of allergenic components in individual profiles of patients.
